# Clinical significance of platelet-to-white blood cell ratio in patients with Wilson disease: a retrospective cohort study

**DOI:** 10.7717/peerj.19379

**Published:** 2025-04-29

**Authors:** Hao-Jie Zhong, Jun-Yi Chen, Wei-Ming Wu, Xing-Xiang He, Yong-Qiang Zhan

**Affiliations:** 1 Shenzhen Second People’s Hospital, Shenzhen, China; 2 The First Affiliated Hospital of Guangdong Pharmaceutical University, Guangzhou, China

**Keywords:** Blood platelets, Hepatolenticular degeneration, Leukocytes, Liver cirrhosis, Splenectomy

## Abstract

**Objective:**

To assess the correlation between the platelet-to-white blood cell ratio (PWR) and the severity of liver dysfunction, hepatic complications, and prognosis in Wilson disease (WD) patients.

**Methods:**

A retrospective analysis was conducted on medical records from January 1, 2016, to March 30, 2022. Both univariate and multivariate analyses were performed to examine the impact of a low PWR (<26.3) on WD severity, liver complications, and disease progression. Additionally, the effect of splenectomy on PWR was evaluated.

**Results:**

The study included 315 patients with WD, among whom 105 had a low PWR and 210 had a high PWR. Those with low PWR exhibited significantly elevated levels of bilirubin, international normalized ratio, prothrombin time, procollagen type-III N-terminal propeptide, type IV collagen, hyaluronic acid, and portal vein diameter. Conversely, they had lower levels of albumin, total cholesterol, low-density lipoprotein cholesterol, and triglycerides (all *P* < 0.05). A low PWR correlated with a greater incidence of splenomegaly/hypersplenism, esophagogastric varices, and ascites (all *P* < 0.05). Furthermore, low PWR independently predicted hepatic decompensation (*P* < 0.05), and splenectomy led to a marked increase in PWR among WD patients (*P* < 0.001).

**Conclusion:**

A low PWR in WD patients is linked to heightened disease severity, increased risk of liver complications, and rapid progression to decompensation. The results imply that splenectomy, by enhancing PWR, may serve as a viable strategy to slow WD progression.

## Introduction

Wilson disease (WD, OMIM 277900) is a hereditary metabolic disorder that disrupts copper homeostasis due to mutations in the *ATP7B* gene, which encodes the ATPase copper-transporting β protein (Shribman et al., 2022). While symptomatic WD is uncommon, with an incidence of 1 in 30,000 individuals, the genetic form, defined by the presence of two pathogenic *ATP7B* alleles, is more prevalent, affecting approximately 1 in 7,000 individuals (Coffey et al., 2013; Czlonkowska et al., 2018).

In WD, a disorder where the liver plays a central role in copper metabolism, copper accumulation within the organ typically triggers liver damage as the first apparent manifestation (Fernando et al., 2020). As the condition progresses, liver damage swiftly advances to cirrhosis (Roberts & Socha, 2017), which is present in around 50% of cases at the time of diagnosis (Ferenci et al., 2019). With further progression to decompensated cirrhosis, patients often face life-threatening complications such as hepatic encephalopathy and liver failure (Mulligan & Bronstein, 2020), significantly increasing mortality risk (Czlonkowska et al., 2018). Liver characteristics in WD are notably distinct from those in other liver conditions (Zhong et al., 2019, 2022). While numerous clinical markers are employed to assess the severity and forecast the prognosis of chronic liver diseases (Colapietro et al., 2024; Elkrief et al., 2023; Hernaez et al., 2023; Sanyal et al., 2023), their applicability and reliability in WD remain uncertain.

A growing body of research has highlighted the potential of inflammatory biomarkers derived from blood cell counts, such as the platelet-to-white blood cell (WBC) ratio (PWR), lymphocyte-to-monocyte, and neutrophil-to-lymphocyte ratios, owing to their affordability and practicality (Adamstein et al., 2023, 2021; Liu et al., 2023). Among these, the PWR has emerged as a reliable marker for evaluating disease severity and predicting outcomes in various liver conditions, including hepatic abscess, acute-on-chronic liver failure, and cirrhosis (Kim et al., 2022; Ko et al., 2022; Liu et al., 2020). This study, therefore, aims to explore the relationship between PWR and disease severity, liver complications, and prognosis in patients with WD.

## Methods

### Participants

This study comprised a consecutive cohort of inpatients diagnosed with WD at the First Affiliated Hospital of Guangdong Pharmaceutical University from January 1, 2016, to March 30, 2022. Exclusion criteria included liver diseases of alternative etiologies, patients with pre-existing malignancies, and cases with incomplete medical records.

### Data collection

Clinical data obtained from the electronic medical record system included demographic details, smoking and alcohol consumption history, medical background, duration of WD, anti-copper therapy, urinary copper levels, presence of Kayser–Fleischer rings, neuropsychiatric symptoms, platelet and WBC counts, liver injury markers (alanine transaminase (ALT), aspartate transaminase (AST), and total bilirubin), synthetic function markers (albumin, total cholesterol, high-density lipoprotein cholesterol (HDL-C), and low-density lipoprotein cholesterol (LDL-C)), triglyceride levels, coagulation markers (international normalized ratio (INR) and prothrombin time (PT)), liver fibrosis markers (procollagen type III terminal propeptide (PIIINP), type IV collagen, hyaluronic acid, and laminin), portal vein diameter, cirrhosis status, hepatic complications (splenomegaly/splenectomy, esophagogastric varices, ascites, spontaneous bacterial peritonitis (SBP), renal impairment, portal vein thrombosis, hepatic encephalopathy, liver failure, and hepatocellular carcinoma), Child–Pugh score, and hepatic decompensation. The follow-up period for compensated patients extended from baseline to either hepatic decompensation or the study’s conclusion.

### Methodology of laboratory measurements

Urinary copper concentrations were determined *via* ICP-MS at DAAN Clinical Laboratory Central (Guangzhou, China). Serum PIIINP, type IV collagen, hyaluronic acid, and laminin levels were assessed using an automated chemiluminescence analyzer at the same facility. Platelet and WBC counts were obtained with a blood routine testing instrument (Mindray BC-6900). Standard laboratory techniques on an auto-analyzer (BECKMAN AU5800) were employed to measure serum ALT, aspartate transaminase, total bilirubin, albumin, total cholesterol, HDL-C, LDL-C, and triglycerides. PT and INR were measured and calculated using an automated coagulation analyzer (STAGO STA-R).

### Definitions

In China, the definition of WD aligns with international diagnostic criteria (Czlonkowska et al., 2018), incorporating factors such as age at onset, liver and neurological symptoms, copper concentrations in serum, liver, and urine, serum ceruloplasmin levels, the presence of Kayser–Fleischer rings, and genetic testing (Wang et al., 2023). The PWR is derived by dividing the platelet count by the WBC count, with values in the lowest third (26.3) serving as the threshold. Alcoholism is defined as a weekly alcohol intake exceeding 70 g. Diagnoses for conditions like splenomegaly/splenectomy, ascites, portal vein thrombosis, and hepatocellular carcinoma rely on a combination of physical examination, imaging, pathological assessment, and patient history. Esophagogastric varices are identified through gastroscopy or imaging. SBP is diagnosed when the neutrophil count in ascitic fluid exceeds 250/mm^3^ without a surgically treatable cause within the abdomen. Renal impairment is identified when blood creatinine exceeds 132.6 μmol/L (Piano et al., 2013). Hepatic encephalopathy diagnosis follows the West Haven Classification (Riordan & Williams, 1997), while liver failure is indicated by an INR greater than 1.5 in conjunction with hepatic encephalopathy (Driever et al., 2021). Hepatic decompensation is diagnosed in the presence of ascites, varices, or hepatic encephalopathy (Jachs et al., 2022). The Child–Pugh classification is determined as described previously (Pugh et al., 1973).

### Statistical analyses

The sample size was calculated using the online Power and Sample Size Calculators. Statistical analysis was performed with SPSS version 22 (IBM, Armonk, NY, USA), GraphPad Prism version 8.0.2, and R version 4.3.2. Categorical data were presented as frequencies (percentages), while continuous data were reported as means ± standard deviation for normally distributed data or as medians (interquartile range) for non-normally distributed data. Group comparisons were conducted using chi-square tests, Fisher’s exact tests, unpaired and paired Student’s t-tests, and Mann–Whitney U tests, depending on the data characteristics. Correlations were assessed using Pearson or Spearman methods, based on the distribution of the data.

The study examined the associations between PWR and various liver function parameters and complications using multivariate linear and logistic regression analyses. These models controlled for variables including age, sex, BMI, smoking status, alcohol use, hypertension, diabetes, and anti-copper therapy. To evaluate the diagnostic utility of platelet count, WBC count, and PWR in hepatic decompensation, receiver operating characteristic (ROC) curves were applied to assess specificity and sensitivity. Furthermore, Kaplan–Meier survival analysis and Cox regression models, both unadjusted and adjusted for factors such as age, sex, BMI, alcohol use, smoking, hypertension, diabetes, and anti-copper therapy, were employed to determine if low PWR was an independent risk factor for hepatic decompensation in WD patients. Results from the linear and logistic regressions were presented as beta coefficients with standard errors and odds ratios (ORs) with 95% confidence intervals (CIs), while Cox regression findings were reported as hazard ratios (HRs) with 95% CIs. A *P* value of less than 0.05 was considered statistically significant.

## Results

### Patient characteristics

A total of 320 WD patients were initially enrolled in the study. Following the application of exclusion criteria, which removed one patient with hepatitis B and four with malignant tumors, 315 WD patients remained for analysis. These patients were divided into two groups: 105 with a low PWR (≤26.3) and 210 with a high PWR (>26.3). Baseline characteristics for each group were presented in Table 1. *ATP7B* gene analysis was performed on 19 patients, all of whom displayed mutations.

**Table 1:** Patient characteristics.

	PWR ≤ 26.3 (*n* = 105)	PWR > 26.3 (*n* = 210)	*P* value
Age (years)	25.00 (19.00–32.00)	24.00 (18.00–31.00)	0.134
Male sex	53 (50.48)	110 (52.38)	0.750
BMI (kg/m2)	19.87 (18.37–22.75) (*n* = 93)	19.77 (18.01–21.98) (n = 204)	0.246
Current smoking	9 (8.57)	11 (5.24)	0.253
Alcoholism	2 (1.90)	1 (0.48)	0.538
Hypertension	1 (0.95)	2 (0.95)	1.000
Type 2 diabetes	0 (0)	1 (0.48)	1.000
Age at diagnosis (years)	21.00 (15.50–26.50)	17.00 (10.75–22.00)	<0.001
Time from diagnosis to inclusion in the study (months)	0.00 (0.00–11.00)	0.00 (0.00-10.50)	0.253
Urinary copper (μg/24 h)	690.30 (233.04–1,632.57) (*n* = 53)	526.78 (199.89–921.60) (*n* = 104)	0.215
Kayser–Fleischer rings	45 (42.86)	58 (27.62)	0.007
Neurological manifestation	73 (69.52)	126 (60.00)	0.099
Psychiatric manifestation	12 (11.43)	22 (10.48)	0.797
Jaundice	8 (7.62)	4 (1.90)	0.029
Clinical forms			0.056
Hepatic	27 (25.71)	79 (37.62)	
Neuropsychiatric	6 (5.71)	19 (9.05)	
Mixed	71 (67.62)	108 (51.43)	
Asymptomatic	1 (0.95)	4 (1.90)	
Anti-copper treatment	77 (73.33)	170 (80.95)	0.121
Anti-copper drugs	*n* = 77	*n* = 170	0.522
Sodium dimercaptosulfonate	59 (76.62)	133 (78.24)	
Penicillamine	3 (3.90)	10 (5.88)	
Dimercaptosuccinic acid	0 (0)	2 (1.18)	
Sodium dimercaptosulfonate + penicillamine	15 (19.48)	22 (12.94)	
Sodium dimercaptosulfonate + dimercaptosuccinic acid	0 (0)	2 (1.18)	
Penicillamine + Dimercaptosuccinic acid	0 (0)	1 (0.59)	
Treatment time (years)	21.00 (15.50–26.00) (*n* = 77)	16.00 (10.00–22.00) (*n* = 170)	<0.001

**Note:**

Categorical data are presented as frequencies (percentages), while continuous data are reported as means ± standard deviation for normally distributed variables or as medians (interquartile range) for non-normally distributed variables. BMI, body mass index; PWR, platelet-to-white blood cell ratio.

### Low PWR is associated with WD-related liver dysfunction

In patients with WD, those exhibiting low PWR demonstrated significantly higher ALT and total bilirubin levels compared to those with high PWR (*P* < 0.05 for both). With respect to synthetic function, the low PWR group showed markedly lower albumin, total cholesterol, LDL-C, and triglyceride levels (*P* < 0.01 for all). Coagulation parameters were also impaired in the low PWR group, which had substantially elevated INR and PT values relative to the high PWR group (*P* < 0.001 for both). Concerning liver fibrosis markers, concentrations of type IV collagen and hyaluronic acid were significantly higher in the low PWR group (*P* < 0.001 for both). Additionally, patients with WD and low PWR exhibited notably larger portal vein diameters and a higher prevalence of cirrhosis (*P* < 0.01 for both, Table 2). These trends were consistent across subgroups of male, female, and untreated patients (Tables S1–S3).

**Table 2:** Association of PWR (cut-off: 26.3) with liver function parameters in patients with WD.

	PWR ≤ 26.3 (*n* = 105)	PWR > 26.3 (*n* = 210)	*P* value
Liver injury parameters			
ALT (U/L)	28.00 (22.00–45.50) (*n* = 105)	25.00 (18.00–42.00) (*n* = 207)	0.013
AST (U/L)	30.00 (18.50–48.10) (*n* = 105)	27.00 (17.00–45.00) (*n* = 207)	0.262
Total bilirubin (μmol/L)	15.30 (9.65–24.95) (*n* = 105)	10.30 (7.80–14.60) (*n* = 207)	<0.001
Synthetic function parameters			
Albumin (g/L)	38.10 (35.00–42.00) (*n* = 105)	40.00 (37.00–43.00) (*n* = 207)	0.002
Total cholesterol (mmol/L)	3.62 (2.98–4.17) (*n* = 88)	3.97 (3.54–4.66) (*n* = 177)	<0.001
HDL-C (mmol/L)	1.21 (0.99–1.40) (*n* = 88)	1.26 (1.07–1.47) (*n* = 177)	0.085
LDL-C (mmol/L)	2.02 (1.57–2.29) (*n* = 88)	2.26 (1.77–2.64) (*n* = 177)	0.002
Triglyceride (mmol/L)	0.81 (0.62–1.06) (*n* = 88)	0.97 (0.71–1.34) (*n* = 177)	0.003
Coagulation parameters			
INR	1.18 (1.10–1.44) (*n* = 101)	1.07 (1.00–1.13) (*n* = 196)	<0.001
PT (s)	14.90 (14.15–17.35) (*n* = 101)	13.90 (13.10–14.68) (*n* = 196)	<0.001
Liver fibrosis parameters			
PIIINP (μg/mL)	82.89 (64.53–116.21) (*n* = 90)	75.96 (54.79–107.94) (*n* = 182)	0.141
Type IV collagen (ng/mL)	63.45 (53.67–76.09) (*n* = 94)	53.84 (45.76–62.12) (*n* = 184)	<0.001
Hyaluronic acid (ng/mL)	87.86 (54.72–199.74) (*n* = 94)	39.21 (25.86–70.28) (*n* = 184)	<0.001
Laminin (ng/mL)	107.19 (97.05–118.27) (*n* = 94)	110.95 (97.91–124.29) (*n* = 184)	0.256
Portal vein diameter (mm)	10.00 (9.00–12.00) (*n* = 80)	10.00 (8.00–11.00) (*n* = 144)	0.005
Cirrhosis	89 (84.76)	131 (62.38)	<0.001

**Note:**

Categorical data are presented as frequencies (percentages), while continuous data are reported as means ± standard deviation for normally distributed variables or as medians (interquartile range) for non-normally distributed variables. ALT, alanine transaminase; AST, aspartate transaminase; BMI, body mass index; HDL-C, high-density lipoprotein cholesterol; INR, international normalized ratio; LDL-C, low-density lipoprotein cholesterol; PIIINP, procollagen type III terminal propeptide; PT, prothrombin time; PWR, platelet-to-white blood cell ratio; WD, Wilson disease

After adjusting for potential confounders, multivariate linear regression revealed that patients with low PWR exhibited significantly higher levels of total bilirubin, INR, PT, PIIINP, type IV collagen, hyaluronic acid, and portal vein diameter. In contrast, these patients had notably lower concentrations of albumin, total cholesterol, LDL-C, and triglycerides compared to those with high PWR (*P* < 0.05 for all parameters, Table 3).

**Table 3:** Multivariate linear regression analyses of low PWR (< 26.3) as a risk factor for worse liver function in patients with WD.

	Beta	Standard error	*P* value
Liver injury parameters			
ALT (U/L)	–	–	–
AST (U/L)	–	–	–
Total bilirubin (μmol/L)	8.83	2.10	<0.001
Synthetic function parameters			
Albumin (g/L)	−1.97	0.58	0.001
Total cholesterol (mmol/L)	−0.48	0.12	<0.001
HDL-C (mmol/L)	–	–	–
LDL-C (mmol/L)	−0.33	0.11	0.003
Triglyceride (mmol/L)	−0.24	0.11	0.024
Coagulation parameters			
INR	0.26	0.04	<0.001
PT (s)	2.25	0.34	<0.001
Liver fibrosis parameters			
PIIINP (μg/mL)	12.63	6.19	0.042
Type IV collagen (ng/mL)	11.70	2.46	<0.001
Hyaluronic acid (ng/mL)	97.27	20.22	<0.001
Laminin (ng/mL)	–	–	–
Portal vein diameter (mm)	0.87	0.27	0.002

**Note:**

Analyses were adjusted for age, sex, BMI, smoking, alcoholism, hypertension, diabetes, and anti-copper treatment. ALT, alanine transaminase; AST, aspartate transaminase; BMI, body mass index; HDL-C, high-density lipoprotein cholesterol; INR, international normalized ratio; LDL-C, low-density lipoprotein cholesterol; PIIINP, procollagen type III terminal propeptide; PT, prothrombin time; PWR, platelet-to-white blood cell ratio; WD, Wilson disease

Correlation analysis further demonstrated that PWR was negatively associated with ALT, total bilirubin, INR, PT, type IV collagen, hyaluronic acid, and portal vein diameter, while it showed positive correlations with albumin, total cholesterol, LDL-C, triglycerides, and laminin in patients with WD (*P* < 0.05 for all, Table 4).

**Table 4:** Spearman correlations between PWR and liver function parameters in patients with WD.

	R	*P* value
Liver injury parameters		
ALT (U/L)	−0.12	0.039
AST (U/L)	−0.05	0.361
Total bilirubin (μmol/L)	−0.33	<0.001
Synthetic function parameters		
Albumin (g/L)	0.20	<0.001
Total cholesterol (mmol/L)	0.25	<0.001
HDL-C (mmol/L)	0.10	0.108
LDL-C (mmol/L)	0.19	0.002
Triglyceride (mmol/L)	0.23	<0.001
Coagulation parameters		
INR	−0.51	<0.001
PT (s)	−0.47	<0.001
Liver fibrosis parameters		
PIIINP (μg/mL)	−0.11	0.066
Type IV collagen (ng/mL)	−0.30	<0.001
Hyaluronic acid (ng/mL)	−0.46	<0.001
Laminin (ng/mL)	0.13	0.037
Portal vein diameter (mm)	−0.28	<0.001

**Note:**

ALT, alanine transaminase; AST, aspartate transaminase; BMI, body mass index; HDL-C, high-density lipoprotein cholesterol; INR, international normalized ratio; LDL-C, low-density lipoprotein cholesterol; PIIINP, procollagen type III terminal propeptide; PT, prothrombin time; PWR, platelet-to-white blood cell ratio; WD, Wilson disease

### Low PWR is a risk factor for WD-related liver complications

Patients with WD and low PWR demonstrated a significantly greater prevalence of splenomegaly or splenectomy (66.67% *vs*. 50.48%, *P* < 0.01), esophagogastric varices (13.33% *vs*. 1.43%, *P* < 0.001), ascites (14.29% *vs*. 0.48%, *P* < 0.001), hepatic encephalopathy (4.76% *vs*. 0.48%, *P* < 0.05), liver failure (4.76% *vs*. 0%, *P* < 0.01), advanced Child–Pugh classification (26.73% *vs*. 2.58%, *P* < 0.001), and hepatic decompensation (23.81% *vs*. 2.38%, *P* < 0.001, Table 5) compared to those with high PWR. Subgroup analysis further identified that low PWR was linked to a higher incidence of WD-related liver complications in both male and female patients (Tables S4 and S5).

**Table 5:** Association of PWR (cut-off: 26.3) with WD-related hepatic complications.

	PWR ≤ 26.3 (*n* = 105)	PWR > 26.3 (*n* = 210)	*P* value
Splenomegaly/splenectomy	70 (66.67)	106 (50.48)	0.006
Esophagogastric varices	14 (13.33)	3 (1.43)	<0.001
Ascites	15 (14.29)	1 (0.48)	<0.001
SBP	1 (0.95)	0 (0)	0.333
Renal impairment	1 (0.95)	0 (0)	0.333
Portal vein thrombosis	0 (0)	1 (0.48)	1.000
Hepatic encephalopathy	5 (4.76)	1 (0.48)	0.029
Liver failure	5 (4.76)	1 (0.48)	0.007
Hepatocellular carcinoma	0 (0)	0 (0)	–
Child–Pugh classification	(*n* = 101)	(*n* = 194)	<0.001
A	74 (73.27)	189 (97.42)	
B/C	27 (26.73)	5 (2.58)	
Hepatic decompensation	25 (23.81)	5 (2.38)	<0.001

**Note:**

Data are presented as n (%). PWR, platelet-to-white blood cell ratio; SBP, spontaneous bacterial peritonitis; WD, Wilson disease.

Logistic regression analyses revealed that low PWR independently predicted several complications in WD patients. Specifically, low PWR was associated with an increased risk of splenomegaly/splenectomy (OR = 1.93, 95% CI [1.15–3.25], *P* < 0.05), esophagogastric varices (OR = 9.93, 95% CI [2.73–36.10], *P* < 0.001), ascites (OR = 21.75, 95% CI [2.71–174.37], *P* < 0.01), and hepatic decompensation (OR = 9.71, 95% CI [3.47–27.15], *P* < 0.001; Table 6).

**Table 6:** Logistic regression analyses of decreased PWR (<26.3) as a risk factor for WD-related hepatic complications.

	OR	95% CI	*P* value
Splenomegaly/splenectomy	1.93	[1.15–3.25]	0.013
Esophagogastric varices	9.93	[2.73–36.10]	<0.001
Ascites	21.75	[2.71–174.37]	0.004
SBP	–	–	–
Renal impairment	–	–	–
Portal vein thrombosis	–	–	–
Hepatic encephalopathy	–	–	–
Liver failure	–	–	–
Hepatocellular carcinoma	–	–	–
Advanced Child–Pugh classification	–	–	–
Hepatic decompensation	9.71	[3.47–27.15]	<0.001

**Note:**

CI, confidence interval; OR, odds ratio; PWR, platelet-to-white blood cell ratio; SBP, spontaneous bacterial peritonitis; WD, Wilson disease.

**Figure 1:**
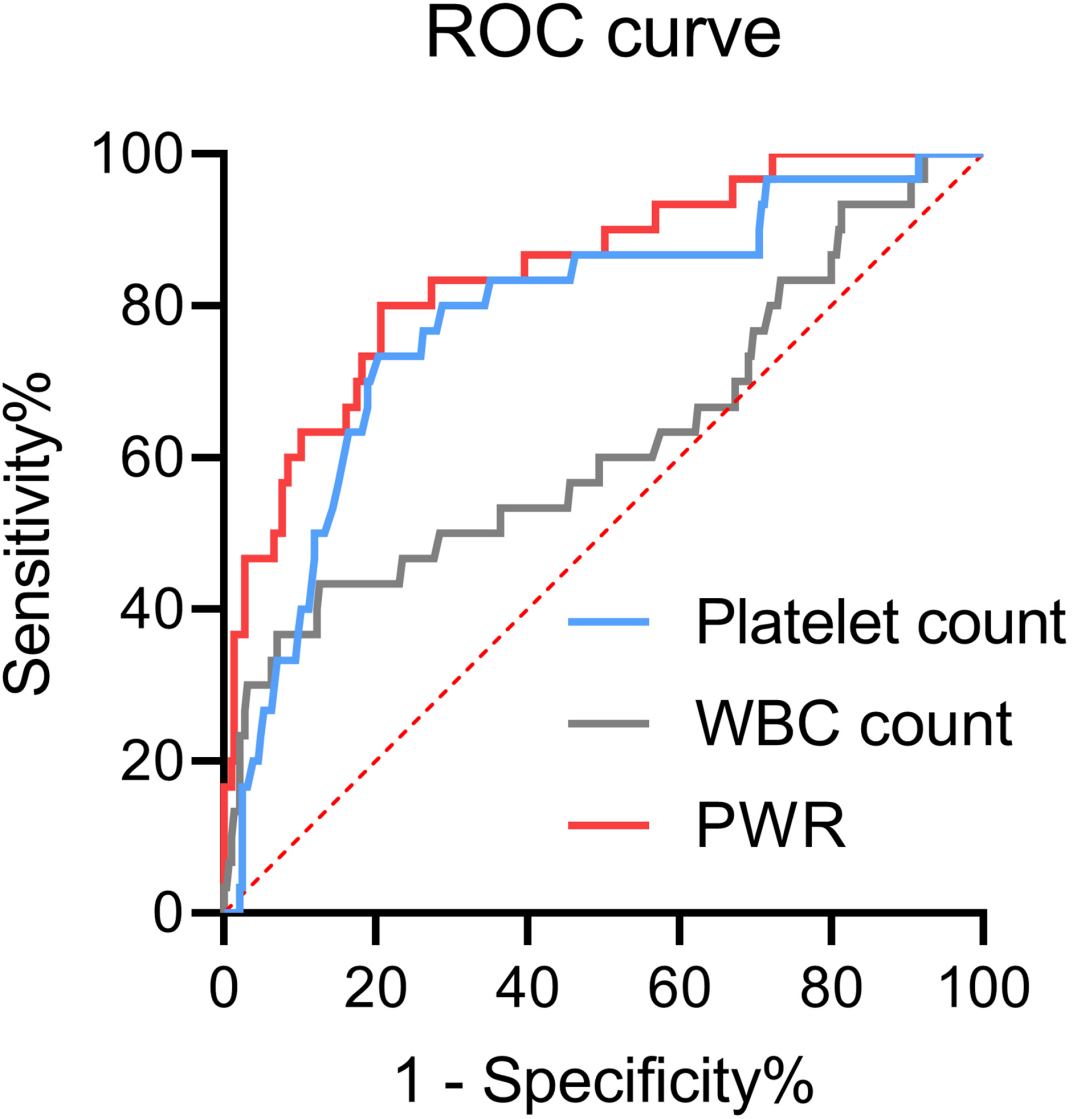
Receiver operating characteristic (ROC) curves of platelets, WBCs, or PWR for hepatic decompensation in patients with WD. PWR, platelet-to-white blood cell ratio; WBC, white blood cell; WD, Wilson disease.

Figure 1 illustrated the use of ROC curves to assess the diagnostic value of platelet count, WBC count, and PWR in differentiating hepatic decompensation from compensation. The area under the curve (AUC) values were 0.85 (95% CI [0.77–0.92]) for PWR, 0.78 (95% CI [0.69–0.87]) for platelet count, and 0.62 (95% CI [0.50–0.74]) for WBC count. The DeLong test confirmed that the AUC for PWR was significantly greater than for both platelet and WBC counts (*P* < 0.05 for each comparison).

### Baseline low PWR is an independent risk factor for predicting hepatic decompensation during follow-up

Among the 285 WD patients initially presenting with hepatic compensation, 234 completed at least one follow-up visit. Over a median follow-up duration of 34.78 months (range: 15.18–50.58), 18 patients progressed to hepatic decompensation. Kaplan–Meier analysis revealed a more rapid progression to decompensation in those with low PWR (*P* < 0.01). After multivariable adjustments, the Cox proportional hazards model identified low PWR as an independent predictor of hepatic decompensation, with a hazard ratio of 3.68 (95% CI [1.21–11.18], *P* < 0.05).

### Splenectomy is an effective approach to recover PWR

**Figure 2:**
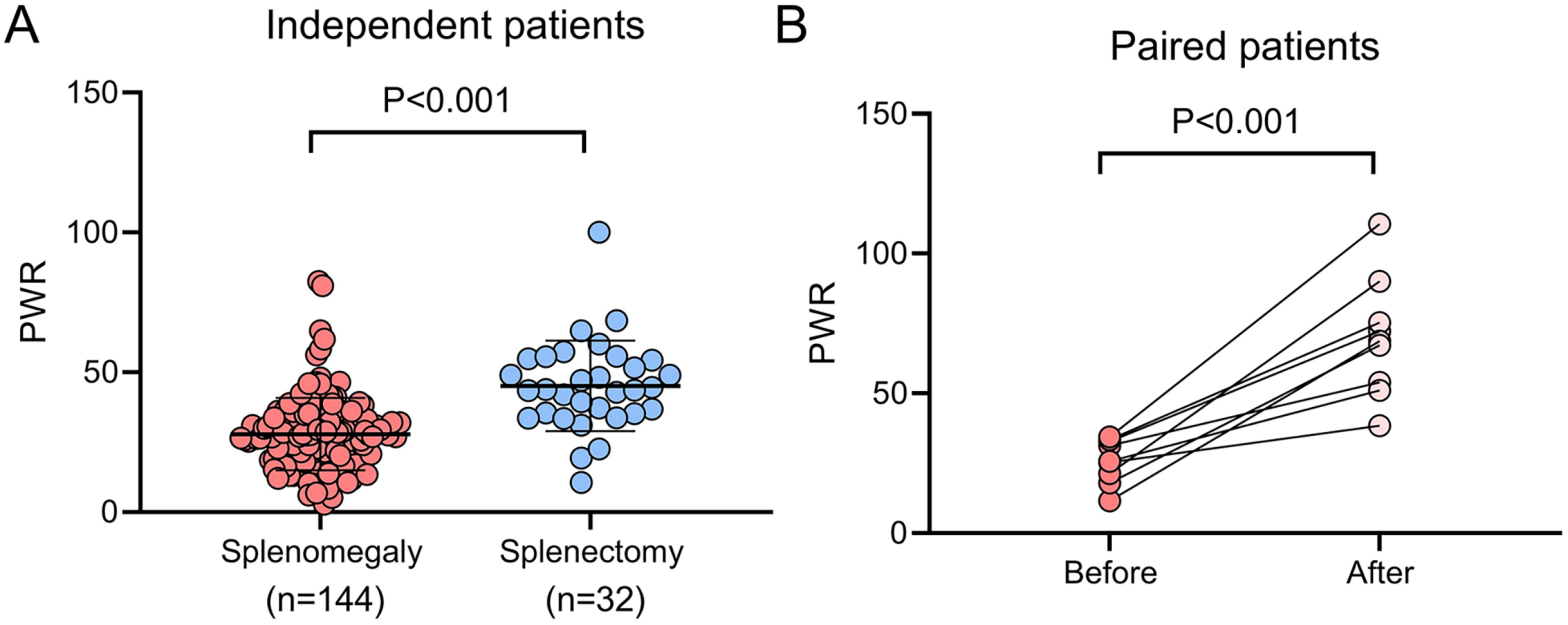
Impact of splenectomy on PWR in patients with WD. (A) Comparison of PWR between WD patients with splenomegaly who underwent splenectomy (*n* = 32) and those who did not (*n* = 144). (B) Pre-and post-splenectomy PWR analysis in WD patients (*n* = 9) PWR, platelet-to-white blood cell ratio; WD, Wilson disease.

Hypersplenism frequently contributes to thrombocytopenia in WD patients (Strickland, Chang & Beckner, 1972), prompting an investigation into the effect of splenectomy on PWR in this cohort. Compared to patients with splenomegaly who did not undergo splenectomy, those who underwent the procedure demonstrated significantly higher platelet counts (118.00 [80.00–168.25] *vs*. 282.00 [221.25–338.25], *P* < 0.001), WBC counts (4.55 [3.53–5.71] *vs*. 6.24 [4.94–8.04], *P* < 0.001), and PWR values (27.00 [18.56–34.46] *vs*. 43.70 [35.21–54.69], *P* < 0.001, Fig. 2A). Additionally, a subgroup of nine patients who had not initially undergone splenectomy but did so during the follow-up period showed significant increases in platelet count (95.33 ± 51.39 *vs*. 439.33 ± 163.96, *P* < 0.001), WBC count (2.88 [2.32–4.62] *vs*. 6.03 [4.77–7.44], *P* < 0.05), and PWR (25.99 ± 7.88 *vs*. 69.72 ± 21.52, *P* < 0.001, Fig. 2B) after splenectomy.

## Discussion

This study establishes a clear association between low PWR and increased WD severity, marked by more severe liver injury and fibrosis, impaired synthetic and coagulative functions, and elevated portal vein pressure. Additionally, low PWR correlates with a higher risk of hepatic complications, including splenomegaly/hypersplenism, esophagogastric varices, and ascites, as well as a more rapid progression to decompensation. The results further suggest that splenectomy effectively restores PWR in WD patients. To our knowledge, this is the first investigation to explore the relationship between PWR and the severity, complications, and progression of WD.

Two potential explanations may account for these results. First, severe chronic liver disease typically induces hypersplenism through portal hypertension and reduced hepatic thrombopoietin production, leading to increased platelet clearance and diminished platelet production (Lim & Cuker, 2022). Furthermore, a reduced platelet count correlates with greater disease severity and poorer outcomes in patients with acute-on-chronic liver failure and hepatocellular carcinoma (Ouyang et al., 2021; Peng et al., 2021; Schrecker et al., 2022). Second, an elevated WBC count in liver disease patients often signals significant inflammation due to liver damage and may indicate a concurrent infection. Such elevations are linked to more severe disease progression and worse prognosis in conditions like non-alcoholic fatty liver disease and acute-on-chronic liver failure (Wang et al., 2016; Yu et al., 2024). Consequently, the PWR may serve as a more reliable and robust marker for evaluating the severity and prognosis of WD.

Multiple studies have identified PWR as a reliable biomarker for assessing the severity of various diseases (Amalia & Dalimonthe, 2020; Tiuca et al., 2023). For example, a multicenter study by Ko et al. (2022), involving 833 patients with pyogenic liver abscesses, found that patients with low PWR (<17.05) exhibited significantly higher ALT levels and lower albumin compared to those with high PWR. Similarly, a prospective cohort study by Kim et al. (2022), which included 1,670 patients with acute decompensation of cirrhosis, reported that a low PWR (≤12.1) correlated with notably lower albumin levels, as well as elevated INR and Child-Pugh scores. Jie et al. (2018) also demonstrated that a low PWR (<9.0) was linked to elevated ALT, total bilirubin, and MELD scores in patients with acute-on-chronic liver failure. Consistent with these findings, our study revealed that a low PWR was associated with more severe liver dysfunction, advanced fibrosis, and increased portal vein pressure in WD patients. Although gender has been associated with the clinical presentation of WD (Ferenci et al., 2019), our results show that both male and female patients with low PWR exhibit more pronounced liver dysfunction. This supports the notion that low PWR reflects liver damage independent of gender. Additionally, PWR was inversely correlated with liver injury, coagulation, and fibrosis markers, while positively correlating with synthetic function parameters, further emphasizing PWR’s role as a key indicator of WD severity.

This study identifies low PWR as a significant risk factor for various hepatic complications, including splenomegaly/hypersplenism, esophagogastric varices, and ascites, in patients with WD. Given their association with portal hypertension, these results suggest that PWR may serve as an important clinical marker for evaluating complications related to portal hypertension. In line with this, Kim et al. (2022) reported that cirrhotic patients with acute deterioration and low PWR had markedly higher incidences of ascites, bacterial infections, gastrointestinal bleeding, and acute-on-chronic liver failure compared to those with high PWR. Ko et al. (2022) similarly found that low PWR correlated with a significantly greater incidence of complications in liver abscess patients. While low PWR was linked to an increased occurrence of liver failure and hepatic encephalopathy in WD patients, further analysis with adjustments revealed that it did not intensify the risk for these complications. Moreover, our analysis demonstrated that patients with low PWR were at an elevated risk of hepatic decompensation compared to those with high PWR. Cox regression further indicated that PWR outperformed platelets and WBC counts as a predictor of decompensation.

Recent studies have identified PWR as a prognostic factor in various liver diseases (Jie et al., 2018; Kim et al., 2022; Ko et al., 2022; Liu et al., 2020; Zhang et al., 2020). A low PWR has been consistently associated with increased mortality in acute-on-chronic liver failure and hepatitis B virus-related decompensated cirrhosis (Jie et al., 2018; Liu et al., 2020; Zhang et al., 2020). Additionally, low PWR serves as a predictor for prolonged hospitalization in liver abscess patients (Ko et al., 2022) and for adverse outcomes, such as liver transplantation or death, in those with cirrhosis experiencing acute deterioration (Kim et al., 2022). In line with these results, our clinical cohort study demonstrated that patients with WD and a low PWR faced a 3.68-fold increased risk of progressing to decompensation compared to those with high PWR, highlighting the importance of low PWR as a key risk factor for decompensation in WD.

Targeting the spleen through splenectomy or splenic embolization has proven effective in increasing platelet counts in patients with WD-related hypersplenism or cirrhosis (Li et al., 2015; Matsukiyo et al., 2018; Yamamoto et al., 2015). Splenectomy, in particular, has demonstrated greater efficacy than splenic embolization in enhancing platelet levels (Huang et al., 2022). The current study revealed that patients with WD who underwent splenectomy experienced significant increases in both platelet counts and PWR, compared to those with splenomegaly who did not receive the procedure. Additionally, within-subject analysis showed a substantial rise in platelet counts and PWR levels post-splenectomy compared to preoperative values. These results suggest that splenectomy-induced improvement in PWR could represent an effective strategy to delay progression to decompensation in WD patients.

Several limitations of this study warrant consideration. First, the exclusive enrollment of hospitalized patients, who typically present at more advanced disease stages, may introduce selection bias into the findings. Second, the incidence of certain complications, such as SBP, renal impairment, portal vein thrombosis, and hepatocellular carcinoma, was either extremely low or absent, limiting the assessment of the relationship between PWR and these complications in WD patients. Third, a substantial proportion of WD patients in this cohort (21.59%) either did not receive anti-copper therapy or discontinued treatment, potentially affecting liver function and the development of complications. Finally, despite including 315 patients, the sample size remains relatively small due to the rarity of WD. Consequently, these findings should be interpreted with caution and further studies are needed for validation.

In conclusion, low PWR correlates with greater disease severity, an increased risk of liver complications, and faster progression to decompensation in WD patients. Splenectomy, by restoring PWR, may offer a potential strategy to delay decompensation progression.

## Supplemental Information

10.7717/peerj.19379/supp-1 Supplemental Information 1 Association of PWR (cut-off: 26.3) with liver function parameters in male patients with WD. Categorical data are presented as frequencies (percentages), while continuous data are reported as means ± standard deviation for normally distributed variables or as medians (interquartile range) for non-normally distributed variables. ALT, alanine transaminase; AST, aspartate transaminase; BMI, body mass index; HDL-C, high-density lipoprotein cholesterol; INR, international normalized ratio; LDL-C, low-density lipoprotein cholesterol; PIIINP, procollagen type III terminal propeptide; PT, prothrombin time; PWR, platelet-to-white blood cell ratio; WD, Wilson disease

10.7717/peerj.19379/supp-2 Supplemental Information 2 Association of PWR (cut-off: 26.3) with liver function parameters in female patients with WD. Categorical data are presented as frequencies (percentages), while continuous data are reported as means ± standard deviation for normally distributed variables or as medians (interquartile range) for non-normally distributed variables. ALT, alanine transaminase; AST, aspartate transaminase; BMI, body mass index; HDL-C, high-density lipoprotein cholesterol; INR, international normalized ratio; LDL-C, low-density lipoprotein cholesterol; PIIINP, procollagen type III terminal propeptide; PT, prothrombin time; PWR, platelet-to-white blood cell ratio; WD, Wilson disease

10.7717/peerj.19379/supp-3 Supplemental Information 3 Association of PWR (cut-off: 26.3) with liver function parameters in untreated patients with WD. Categorical data are presented as frequencies (percentages), while continuous data are reported as means ± standard deviation for normally distributed variables or as medians (interquartile range) for non-normally distributed variables. ALT, alanine transaminase; AST, aspartate transaminase; BMI, body mass index; HDL-C, high-density lipoprotein cholesterol; INR, international normalized ratio; LDL-C, low-density lipoprotein cholesterol; PIIINP, procollagen type III terminal propeptide; PT, prothrombin time; PWR, platelet-to-white blood cell ratio; WD, Wilson disease

10.7717/peerj.19379/supp-4 Supplemental Information 4 Association of PWR (cut-off: 26.3) with WD-related hepatic complications in male patients. Data are presented as n (%). PWR, platelet-to-white blood cell ratio; SBP, spontaneous bacterial peritonitis; WD, Wilson disease.

10.7717/peerj.19379/supp-5 Supplemental Information 5 Association of PWR (cut-off: 26.3) with WD-related hepatic complications in female patients. Data are presented as n (%). PWR, platelet-to-white blood cell ratio; SBP, spontaneous bacterial peritonitis; WD, Wilson disease.

10.7717/peerj.19379/supp-6 Supplemental Information 6 Raw data.

10.7717/peerj.19379/supp-7 Supplemental Information 7 STROBE checklist.

## Additional Information and Declarations

### Data Availability

The following information was supplied regarding data availability:

The raw data supporting these findings are available in the Supplemental File.
