# Supplementary material for: Clinical significance of platelet-to-white blood cell ratio in patients with Wilson disease: a retrospective cohort study
Source: PeerJ. 2025 Apr 29;13:e19379. doi: 10.7717/peerj.19379 (PMC12047222; doi:10.7717/peerj.19379)
Supplement: Supplemental Information 1 — Categorical data are presented as frequencies (percentages), while continuous data are reported as means ± standard deviation for normally distributed variables or as medians (interquartile range) for non-normally distributed variables. ALT, alanine transaminase; AST, aspartate transaminase; BMI, body mass index; HDL-C, high-density lipoprotein cholesterol; INR, international normalized ratio; LDL-C, low-density lipoprotein cholesterol; PIIINP, procollagen type III terminal propeptide; PT, prothrombin time; PWR, platelet-to-white blood cell ratio; WD, Wilson disease [file peerj-13-19379-s001.docx]

**Table S1. Association of PWR (cut-off: 26.3) with liver function parameters in male patients with WD**

|  | PWR ≤26.3 (n = 53) | PWR >26.3 (n = 110) | P value |
| --- | --- | --- | --- |
| Liver injury parameters |  |  |  |
| ALT (U/L) | 28.00 (23.00–43.00)  (n = 53) | 28.00 (20.00–50.00)  (n = 107) | 0.502 |
| AST (U/L) | 35.00 (22.50–50.50)  (n = 53) | 30.00 (20.00–56.00)  (n = 107) | 0.919 |
| Total bilirubin (μmol/L) | 15.70 (9.85–27.00)  (n = 53) | 11.50 (8.10–17.10)  (n = 107) | <0.001 |
| Synthetic function parameters |  |  |  |
| Albumin (g/L) | 38.68±5.29  (n = 53) | 41.20±4.40  (n = 107) | 0.002 |
| Total cholesterol (mmol/L) | 3.73 (3.36–4.29)  (n = 44) | 4.11 (3.65–5.17)  (n = 90) | 0.018 |
| HDL-C (mmol/L) | 1.25±0.31  (n = 44) | 1.26±0.27  (n = 90) | 0.781 |
| LDL-C (mmol/L) | 2.07 (1.76–2.43)  (n = 44) | 2.41 (1.89–2.86)  (n = 90) | 0.022 |
| Triglyceride (mmol/L) | 0.84 (0.65–1.11)  (n = 44) | 1.02 (0.76–1.53)  (n = 90) | 0.030 |
| Coagulation parameters |  |  |  |
| INR | 1.18 (1.11–1.47)  (n = 50) | 1.06 (0.98–1.13)  (n = 103) | <0.001 |
| PT (s) | 15.05 (14.38–17.48)  (n = 50) | 13.80 (13.00–14.70)  (n = 103) | <0.001 |
| Liver fibrosis parameters |  |  |  |
| PⅢNP (μg/mL) | 86.11 (62.61–115.10)  (n = 44) | 82.26 (56.70–109.00)  (n = 96) | 0.729 |
| Type Ⅳ collagen (ng/mL) | 61.83 (52.10–75.39)  (n = 47) | 55.33 (46.19–63.46)  (n = 98) | 0.003 |
| Hyaluronic acid (ng/mL) | 89.45 (52.36–148.00)  (n = 47) | 41.58 (27.21–77.46)  (n = 98) | <0.001 |
| Laminin (ng/mL) | 107.10 (97.44–118.10)  (n = 47) | 112.30 (100.30–125.20)  (n = 98) | 0.082 |
| Portal vein diameter (mm) | 10.00 (9.00–12.00)  (n = 39) | 10.00 (9.00–12.00)  (n = 78) | 0.255 |
| Urinary copper (μg/24h) | 661.17 (200.90–1684.20)  (n = 24) | 445.20 (168.67–1038.75)  (n = 47) | 0.355 |
| Cirrhosis | 44 (83.02) | 67 (60.91) | 0.005 |

Categorical data are presented as frequencies (percentages), while continuous data are reported as means ± standard deviation for normally distributed variables or as medians (interquartile range) for non-normally distributed variables. ALT, alanine transaminase; AST, aspartate transaminase; BMI, body mass index; HDL-C, high-density lipoprotein cholesterol; INR, international normalized ratio; LDL-C, low-density lipoprotein cholesterol; PⅢNP, procollagen type Ⅲ terminal propeptide; PT, prothrombin time; PWR, platelet-to-white blood cell ratio; WD, Wilson disease
