# Supplementary material for: Clinical significance of platelet-to-white blood cell ratio in patients with Wilson disease: a retrospective cohort study
Source: PeerJ. 2025 Apr 29;13:e19379. doi: 10.7717/peerj.19379 (PMC12047222; doi:10.7717/peerj.19379)
Supplement: Supplemental Information 2 — Categorical data are presented as frequencies (percentages), while continuous data are reported as means ± standard deviation for normally distributed variables or as medians (interquartile range) for non-normally distributed variables. ALT, alanine transaminase; AST, aspartate transaminase; BMI, body mass index; HDL-C, high-density lipoprotein cholesterol; INR, international normalized ratio; LDL-C, low-density lipoprotein cholesterol; PIIINP, procollagen type III terminal propeptide; PT, prothrombin time; PWR, platelet-to-white blood cell ratio; WD, Wilson disease [file peerj-13-19379-s002.docx]

**Table S2. Association of PWR (cut-off: 26.3) with liver function parameters in female patients with WD**

|  | PWR ≤26.3 (n = 52) | PWR >26.3 (n = 100) | P value |
| --- | --- | --- | --- |
| Liver injury parameters |  |  |  |
| ALT (U/L) | 31.50 (22.00–48.25) | 22.50 (17.00–34.75) | 0.004 |
| AST (U/L) | 29.00 (17.25–40.75) | 23.00 (15.00–36.00) | 0.100 |
| Total bilirubin (μmol/L) | 15.20 (9.55–22.05) | 9.30 (7.33–13.78) | <0.001 |
| Synthetic function parameters |  |  |  |
| Albumin (g/L) | 37.35 (35.00–41.30) | 39.45 (36.03–42.00) | 0.073 |
| Total cholesterol (mmol/L) | 3.24 (2.79–3.97)  (n = 44) | 3.88 (3.36–4.44)  (n = 87) | 0.001 |
| HDL-C (mmol/L) | 1.17±0.30  (n = 44) | 1.31±0.32  (n = 87) | 0.017 |
| LDL-C (mmol/L) | 1.81 (1.25–2.28)  (n = 44) | 2.17 (1.65–2.44)  (n = 87) | 0.033 |
| Triglyceride (mmol/L) | 0.73 (0.60–1.04)  (n = 44) | 0.92 (0.68–1.24)  (n = 87) | 0.046 |
| Coagulation parameters |  |  |  |
| INR | 1.18 (1.08–1.42)  (n = 51) | 1.08 (1.01–1.13)  (n =93) | <0.001 |
| PT (s) | 14.90 (13.90–17.30)  (n = 51) | 14.10 (13.20–14.65)  (n = 93) | <0.001 |
| Liver fibrosis parameters |  |  |  |
| PⅢNP (μg/mL) | 80.80 (66.60–116.71)  (n = 46) | 71.01 (51.26–104.64)  (n = 86) | 0.082 |
| Type Ⅳ collagen (ng/mL) | 63.47 (55.32–78.24)  (n = 47) | 51.49 (44.44–59.99)  (n = 86) | <0.001 |
| Hyaluronic acid (ng/mL) | 86.56 (56.95–245.45)  (n = 47) | 38.47 (25.57–65.61)  (n = 86) | <0.001 |
| Laminin (ng/mL) | 108.16 (95.89–120.05)  (n = 47) | 108.77 (95.30–122.95)  (n = 86) | 0.882 |
| Portal vein diameter (mm) | 10.00 (9.50–11.00)  (n = 41) | 9.00 (8.00–10.00)  (n = 66) | <0.001 |
| Urinary copper (μg/24h) | 751.36 (251.78–1595.93)  (n = 29) | 595.59 (226.68–908.53)  (n = 57) | 0.415 |
| Cirrhosis | 45 (86.54) | 64 (64.00) | 0.003 |

Categorical data are presented as frequencies (percentages), while continuous data are reported as means ± standard deviation for normally distributed variables or as medians (interquartile range) for non-normally distributed variables. ALT, alanine transaminase; AST, aspartate transaminase; BMI, body mass index; HDL-C, high-density lipoprotein cholesterol; INR, international normalized ratio; LDL-C, low-density lipoprotein cholesterol; PⅢNP, procollagen type Ⅲ terminal propeptide; PT, prothrombin time; PWR, platelet-to-white blood cell ratio; WD, Wilson disease
