# Supplementary material for: Clinical significance of platelet-to-white blood cell ratio in patients with Wilson disease: a retrospective cohort study
Source: PeerJ. 2025 Apr 29;13:e19379. doi: 10.7717/peerj.19379 (PMC12047222; doi:10.7717/peerj.19379)
Supplement: Supplemental Information 3 — Categorical data are presented as frequencies (percentages), while continuous data are reported as means ± standard deviation for normally distributed variables or as medians (interquartile range) for non-normally distributed variables. ALT, alanine transaminase; AST, aspartate transaminase; BMI, body mass index; HDL-C, high-density lipoprotein cholesterol; INR, international normalized ratio; LDL-C, low-density lipoprotein cholesterol; PIIINP, procollagen type III terminal propeptide; PT, prothrombin time; PWR, platelet-to-white blood cell ratio; WD, Wilson disease [file peerj-13-19379-s003.docx]

**Table S3. Association of PWR (cut-off: 26.3) with liver function parameters in untreated patients with WD**

|  | Total (n = 68) | PWR ≤26.3 (n = 28) | PWR >26.3 (n = 40) | P value |
| --- | --- | --- | --- | --- |
| Liver injury parameters |  |  |  |  |
| ALT (U/L) | 28.00 (19.00–47.00)  (n = 67) | 27.50 (22.00–50.50)  (n = 28) | 28.00 (18.00–47.00)  (n = 39) | 0.576 |
| AST (U/L) | 32.00 (14.00–51.00)  (n = 67) | 31.00 (16.25–46.00)  (n = 28) | 32.00 (14.00–56.00)  (n = 39) | 0.972 |
| Total bilirubin (μmol/L) | 10.50 (8.10–18.20)  (n = 67) | 14.55 (9.43–24.60)  (n = 28) | 9.00 (7.20–14.50)  (n = 39) | 0.002 |
| Synthetic function parameters |  |  |  |  |
| Albumin (g/L) | 39.30 (35.20–43.00)  (n = 67) | 35.45 (32.55–42.00)  (n = 28) | 40.00 (37.00–43.00)  (n = 39) | 0.007 |
| Total cholesterol (mmol/L) | 3.89 (3.12–4.39)  (n = 58) | 3.66 (3.14–4.27)  (n = 24) | 3.99 (3.09–4.65)  (n = 34) | 0.382 |
| HDL-C (mmol/L) | 1.16±0.28  (n = 58) | 1.12±0.34  (n = 24) | 1.19±0.22  (n = 34) | 0.440 |
| LDL-C (mmol/L) | 2.23 (1.62–2.65)  (n = 58) | 2.12 (1.70–2.36)  (n = 24) | 2.29 (1.47–2.77)  (n = 34) | 0.352 |
| Triglyceride (mmol/L) | 0.92 (0.72–1.32)  (n = 58) | 0.83 (0.70–1.23)  (n = 24) | 0.99 (0.80–1.41)  (n = 34) | 0.155 |
| Coagulation parameters |  |  |  |  |
| INR | 1.11 (1.03–1.19)  (n = 65) | 1.18 (1.11–1.47)  (n = 27) | 1.07 (1.01–1.13)  (n = 38) | <0.001 |
| PT (s) | 14.20 (13.35–15.00)  (n = 65) | 14.90 (14.20–17.40)  (n = 27) | 13.85 (13.28–14.33)  (n = 38) | <0.001 |
| Liver fibrosis parameters |  |  |  |  |
| PⅢNP (μg/mL) | 83.10 (51.35–117.83)  (n = 61) | 81.30 (46.38–112.28)  (n = 25) | 87.96 (62.93–126.01)  (n = 36) | 0.410 |
| Type Ⅳ collagen (ng/mL) | 63.33 (56.48–76.44)  (n = 61) | 79.18±22.74  (n =25) | 59.33±12.60  (n = 36) | <0.001 |
| Hyaluronic acid (ng/mL) | 74.90 (41.22–150.99)  (n = 61) | 141.42 (76.16–257.69)  (n = 25) | 55.62 (33.73–89.56)  (n = 36) | <0.001 |
| Laminin (ng/mL) | 110.10±22.51  (n = 61) | 108.69 (95.36–120.69)  (n = 25) | 108.27 (94.44–128.83)  (n = 36) | 0.711 |
| Portal vein diameter (mm) | 10.00 (9.00–12.00)  (n = 51) | 10.00 (10.00–12.25)  (n = 26) | 10.00 (9.00–12.00)  (n = 25) | 0.277 |
| Urinary copper (μg/24h) | 766.83 (264.13–1576.00)  (n = 34) | 922.79 (327.06–1928.45)  (n = 14) | 615.64 (206.03–1384.05)  (n = 20) | 0.294 |
| Cirrhosis | 43 (63.24) | 22 (78.57) | 21 (52.50) | 0.028 |

Categorical data are presented as frequencies (percentages), while continuous data are reported as means ± standard deviation for normally distributed variables or as medians (interquartile range) for non-normally distributed variables. ALT, alanine transaminase; AST, aspartate transaminase; BMI, body mass index; HDL-C, high-density lipoprotein cholesterol; INR, international normalized ratio; LDL-C, low-density lipoprotein cholesterol; PⅢNP, procollagen type Ⅲ terminal propeptide; PT, prothrombin time; PWR, platelet-to-white blood cell ratio; WD, Wilson disease
