# Supplementary material for: Clinical significance of platelet-to-white blood cell ratio in patients with Wilson disease: a retrospective cohort study
Source: PeerJ. 2025 Apr 29;13:e19379. doi: 10.7717/peerj.19379 (PMC12047222; doi:10.7717/peerj.19379)
Supplement: Supplemental Information 4 — Data are presented as n (%). PWR, platelet-to-white blood cell ratio; SBP, spontaneous bacterial peritonitis; WD, Wilson disease. [file peerj-13-19379-s004.docx]

**Table S4.** **Association of PWR (cut-off: 26.3) with WD-related hepatic complications in male patients**

|  | PWR ≤26.3  (n = 53) | PWR >26.3  (n = 110) | P value |
| --- | --- | --- | --- |
| Splenomegaly/splenectomy | 37 (69.81) | 55 (50.00) | 0.017 |
| Esophagogastric varices | 7 (13.21) | 3 (2.73) | 0.024 |
| Ascites | 5 (9.43) | 0 (0) | 0.005 |
| SBP | 0 (0) | 0 (0) | - |
| Renal impairment | 0 (0) | 0 (0) | - |
| Portal vein thrombosis | 0 (0) | 1 (0.91) | 1.000 |
| Hepatic encephalopathy | 2 (3.77) | 0 (0) | 0.197 |
| Liver failure | 2 (3.77) | 0 (0) | 0.197 |
| Hepatocellular carcinoma | 0 (0) | 0 (0) | - |
| Child–Pugh classification | (n = 50) | (n = 101) | <0.001 |
| A | 37 (74.00) | 98 (97.03) |  |
| B/C | 13 (26.00) | 3 (2.97) |  |
| Hepatic decompensation | 11 (20.75) | 3 (2.73) | <0.001 |

Data are presented as n (%). PWR, platelet-to-white blood cell ratio; SBP, spontaneous bacterial peritonitis; WD, Wilson disease
