# Supplementary material for: Clinical significance of platelet-to-white blood cell ratio in patients with Wilson disease: a retrospective cohort study
Source: PeerJ. 2025 Apr 29;13:e19379. doi: 10.7717/peerj.19379 (PMC12047222; doi:10.7717/peerj.19379)
Supplement: Supplemental Information 5 — Data are presented as n (%). PWR, platelet-to-white blood cell ratio; SBP, spontaneous bacterial peritonitis; WD, Wilson disease. [file peerj-13-19379-s005.docx]

**Table S5.** **Association of PWR (cut-off: 26.3) with WD-related hepatic complications in female patients**

|  | PWR ≤26.3  (n = 52) | PWR >26.3  (n = 100) | P value |
| --- | --- | --- | --- |
| Splenomegaly/splenectomy | 33 (63.46) | 51 (51.00) | 0.143 |
| Esophagogastric varices | 7 (13.46) | 0 (0) | 0.001 |
| Ascites | 10 (19.23) | 1 (1.00) | <0.001 |
| SBP | 1 (1.92) | 0 (0) | 0.142 |
| Renal impairment | 1 (1.92) | 0 (0) | 0.142 |
| Portal vein thrombosis | 0 (0) | 0 (0) | - |
| Hepatic encephalopathy | 3 (5.77) | 1 (1.00) | 0.227 |
| Liver failure | 3 (5.77) | 0 (0) | 0.070 |
| Hepatocellular carcinoma | 0 (0) | 0 (0) | - |
| Child–Pugh classification | (n = 51) | (n = 93) | <0.001 |
| A | 37 (72.55) | 91 (97.85) |  |
| B/C | 14 (27.45) | 2 (2.15) |  |
| Hepatic decompensation | 14 (26.92) | 2 (2.00) | <0.001 |

Data are presented as n (%). PWR, platelet-to-white blood cell ratio; SBP, spontaneous bacterial peritonitis; WD, Wilson disease
